# TBEV Subtyping in Terms of Genetic Distance

**DOI:** 10.3390/v12111240

**Published:** 2020-10-31

**Authors:** Andrei A. Deviatkin, Galina G. Karganova, Yulia A. Vakulenko, Alexander N. Lukashev

**Affiliations:** 1 Laboratory of Molecular Biology and Biochemistry, Institute of Molecular Medicine, Sechenov First Moscow State Medical University, 119048 Moscow, Russia; alexander_lukashev@hotmail.com; 2 Department of Organization and Technology of Immunobiological Preparations, Institute for Translational Medicine and Biotechnology, Sechenov First Moscow State Medical University, 119991 Moscow, Russia; karganova@bk.ru; 3 Laboratory of Biology of Arboviruses, Chumakov Institute of Poliomyelitis and Viral Encephalitides (FSBSI “Chumakov FSC R&D IBP RAS), 108819 Moscow, Russia; 4 Martsinovsky Institute of Medical Parasitology, Tropical and Vector Borne Diseases, Sechenov First Moscow State Medical University, 119435 Moscow, Russia; vjulia94@gmail.com; 5 Department of Virology, Faculty of Biology, Lomonosov Moscow State University, 119234 Moscow, Russia

**Keywords:** TBEV, taxonomy, subtypes, pairwise genetic distance

## Abstract

Currently, the lowest formal taxon in virus classification is species; however, unofficial lower-level units are commonly used in everyday work. *Tick-borne encephalitis virus* (*TBEV*) is a species of mammalian tick-borne flaviviruses that may cause encephalitis. Many known representatives of *TBEV* are grouped into subtypes, mostly according to their phylogenetic relationship. However, the emergence of novel sequences could dissolve this phylogenetic grouping; in the absence of strict quantitative criterion, it may be hard to define the borders of the first TBEV taxonomic unit below the species level. In this study, the nucleotide/amino-acid space of all known TBEV sequences was analyzed. Amino-acid sequence *p*-distances could not reliably distinguish TBEV subtypes. Viruses that differed by less than 10% of nucleotides in the polyprotein-coding gene belonged to the same subtype. At the same time, more divergent viruses were representatives of different subtypes. According to this distance criterion, *TBEV* species may be divided into seven subtypes: TBEV-Eur, TBEV-Sib, TBEV-FE, TBEV-2871 (TBEV-Ob), TBEV-Him, TBEV-178-79 (TBEV-Bkl-1), and TBEV-886-84 (TBEV-Bkl-2).

## 1. Introduction

Historically, viruses were classified according to phenotypical features, such as virion shape, replication strategy, and host range [1,2]. Recently, the International Committee on Taxonomy of Viruses (ICTV) proposed that viruses should be incorporated into official classification only on the basis of genomic data [1].

Currently, there is no universal system to classify viruses below the species level [3]. Various terms, such as subtype, lineage, clade, group, subgroup, genotype, serotype, and type, among others, may be used for different viruses. The meaning of these terms is not universal among distinct viruses but a commonly accepted convention in each group of researchers, developed decades ago and reproduced ever since [4]. Unification of nomenclature under the species level was proposed for several filoviruses and coronaviruses [3,4], but does not seem to be a universal trend. Notably, according to the current state of viral taxonomy, each of 6590 formally accepted species requires separate and careful consideration at the subspecies level. Thus, a consensus has yet to be reached on the classification of viruses below the species level.

For some viruses, historically accepted terms are used for subspecies classification. Over 50 years ago, Clarke et al. proposed the division of *Tick-borne encephalitis virus* species (*TBEV*) into two distinct subtypes (Russian spring-summer encephalitis and Central European encephalitis) on the basis of serological analyses [5]. The third serotype, Aina/1448, was described in an immunological study published in 1981 [6]. In 1999, phylogenetic analysis demonstrated the segregation of TBE viruses into three subtypes according to their primary geographical distribution: European (Eur), Siberian (Sib), and Far-Eastern (FE) [7]. Hereinafter, we adhere to the traditional term, subtype, to designate the first taxonomic unit below the *TBEV* species level. In recent decades, there has been a growing body of evidence that subtypes can be isolated far from their nominal geographic region [8,9,10,11,12,13,14,15,16,17]. Subspecies taxonomy of *TBEV* was further complicated by the discovery of viruses that could not be unambiguously assigned to known subtypes. In 2001, divergent TBEV strains 178-179 and 886-84 were found in Eastern Siberia near Baikal [18]. These virus groups were provisionally termed genotypes 4 (strain 178–79) and 5 (strain 886-84) [19]. Recently, “886−84-like” viruses were named the “Baikalian subtype” [20,21,22]. The taxonomic position of the sole known “genotype 4” representative, strain 178–79, remains debated [23]. In 2017, a highly divergent strain, TBEV2871 (prospectively termed Obskaya), was found in Western Siberia in the vicinity of the Ob’ river [24]. In 2018, representatives of the Himalayan subtype were described in rodents in the Tibetan Highlands [25]. The status of these novel viruses in terms of subtypes remains uncertain and varies in different publications.

According to the ICTV, species criteria in the *Flavivirus* genus are based on multiple factors, such as sequence data, association with a host, vector, disease, and geographic distribution [26]. Solely phylogenetic data may not be sufficient to distinguish one specific virus from another. For example, the Eur subtype of TBEV (TBEV-Eur) is closer to *Louping ill virus* (*LIV*) than to other TBEV subtypes in terms of genetic distance. Despite this, *LIV* is classified as a separate species according to geographical, pathogenetic, and environmental peculiarities [27]. At the same time, a quantitative criterion for taxonomic assignment at different levels may clarify the relationships among members of the genus *Flavivirus* [28]. Several genetic distance-based species criteria were proposed for the genus *Flavivirus*. Kuno et al. suggested that nucleotide sequence differences over 16% may indicate distinct species [28]. Grard et al. demonstrated a multimodal distribution of amino-acid distances between members of the genus *Flavivirus* that corresponded to intraspecies, intragroup, and intergroup distances [29]. To the best of our knowledge, strict criteria for *TBEV* species demarcation into subtypes remain unclear. Herein, we analyzed all available TBEV sequences to test the possibility of distinguishing TBEV subtypes according to genomic sequence data.

## 2. Materials and Methods

**Figure 1:**
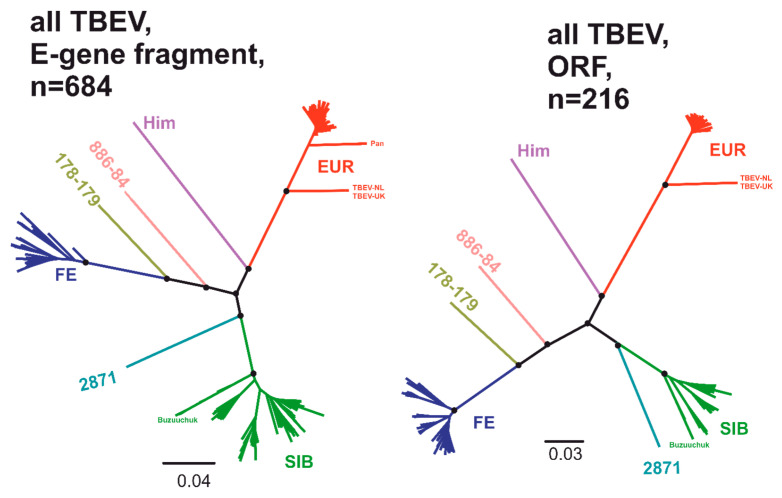
Unrooted maximum likelihood tree for *Tick-borne encephalitis virus* (*TBEV*; *E* gene fragment—left panel, complete open reading frame (ORF)—right panel). Black circles indicate high-level nodes that were supported by UFBoot values over 95% [36]. The scale bar and branch lengths represent the expected number of substitutions per site.

Sequence data were processed as described previously [8] with some modifications. Briefly, all available TBEV sequences represented in GenBank as of July 2020 that contained *E* gene fragment sequences (genome positions 1147–2176 in the reference sequence #NC_001672) were selected (*n* = 987). Alternatively, all available complete open reading frames (ORFs) of TBEV sequences represented in GenBank as of July 2020 were retrieved (*n* = 236 ORFs). Identical sequences were omitted. Synthetic TBEV strain sequences were also excluded. Final datasets consisted of 684 *E* gene fragment (1030 nt) and 216 full ORF (10248 nt) sequences. For further analysis, unrooted maximum likelihood (ML) phylogenetic inference was performed using IQ-TREE [30]. The divergent groups of TBEV (different colors in Figure 1) were extracted into independent datasets. An uncorrected pairwise genetic distance (*p*-distance) distribution was calculated and visualized in the R environment using ape [31], seqinr [32], scales [33], gdata [34], and ggplot [35] packages.

## 3. Results

*TBEV* is distinguished from its nearest sister species, *LIV*, according to the host and pathogenic profile rather than genetic distance [27]. This study did not address species demarcation; thus, *TBEV* was analyzed separately from *LIV*. Classification of a virus ideally requires a full-genome sequence. For some viruses, partial sequences are traditionally used, mainly due to different variation and recombination profiles in distinct genome regions. Even though there have been reports of recombination in TBEV [37], there is no evidence that it is systematic and affects the segregation of subtypes. Thus, a complete ORF sequence was primarily used. There were more than 40 nonidentical full ORF sequences for each of the three major subtypes; however, the number of known sequences for novel provisional subtypes was much smaller (Table 1). In field studies, a fragment of the E protein encoding sequence (colloquially termed the *E*-gene) is commonly used for virus identification; thus, it is important to verify that full ORF criteria are reproduced in the E protein coding sequence. There is no convention regarding the precise borders of the *E* gene fragment used for analysis. For this study, a sequence of 1030 nt (genome positions 1147–2176, according to #NC_001672) was chosen as a tradeoff between sequence length (resolution) and the number of available sequences (Table 1). Unrooted maximum likelihood trees demonstrated that all TBEV sequences segregated into seven groups (different colors in Figure 1) with high UFBoot support values [37]. Further analysis suggested that these seven groups may be regarded as subtypes (first TBEV subspecies taxonomic level), according to the genetic distance distribution.

**Figure 2:**
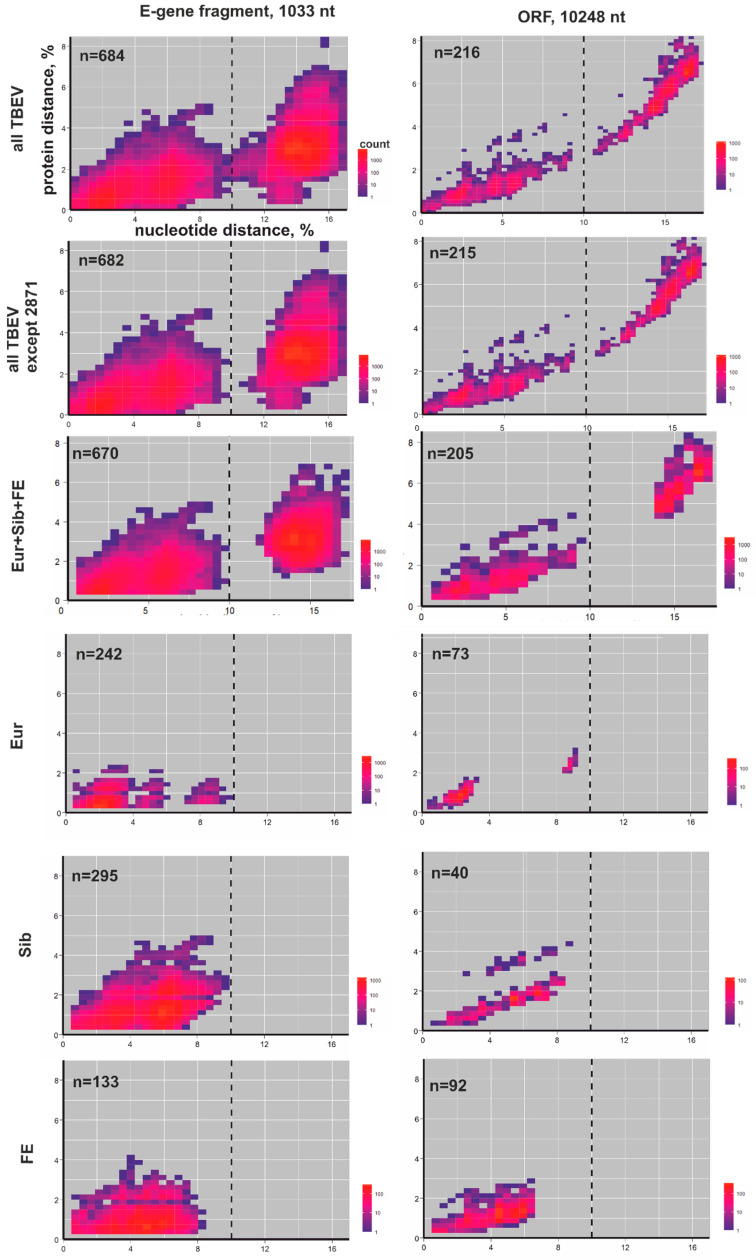
Heatmap of concordance between nucleotide and amino-acid pairwise distances in *E* gene fragments (left panels) and ORF (right panels). First panel: all TBEV sequences. Second panel: all TBEV sequences except “2871” group viruses. Third panel: three major TBEV subtypes. The three bottom panels demonstrate the distribution of intrasubtype virus pairs for TBEV-Eur, TBEV-Sib, and TBEV-FE. Axes show uncorrected amino-acid and nucleotide sequence distances; dots correspond to distances between each possible pair of sequences in the dataset. Color indicates the density of dots according to the scale bar. The dotted line at 10% nucleotide difference indicates the proposed threshold for the division of TBEV species into subtypes.

**Figure 3:**
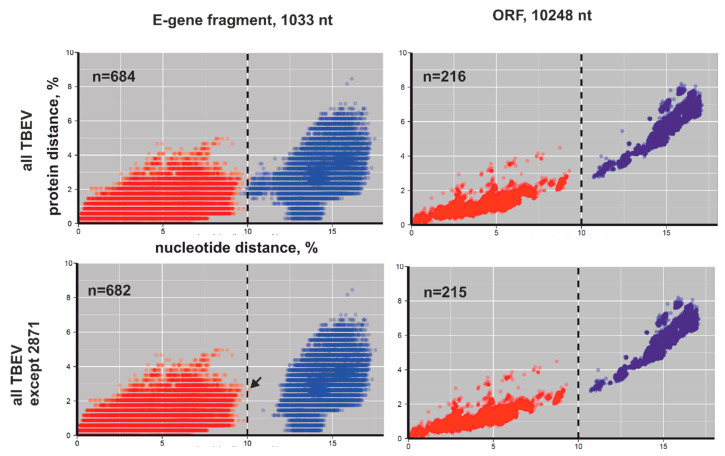
Concordance between nucleotide and amino-acid pairwise distances for TBEV sequences in *E* gene fragment (left panel) and ORF (right panel). First panel: all TBEV sequences. Second panel: all TBEV sequences except “2871” group viruses. Axes show uncorrected amino-acid and nucleotide sequence distances; dots correspond to distances between each possible pair of sequences in the dataset. The dotted line at 10% nucleotide difference indicates the proposed threshold for segregation of TBEV species into subtypes. Virus pairs were divided into intrasubtype pairs (viruses from the same subtype, indicated by the red color) and intersubtype pairs (viruses from different subtypes, indicated by the blue color).

Phylogenetic grouping is a fragile taxonomic criterion since the discovery of additional sequences can affect it dramatically. The distributions of pairwise nucleotide and amino-acid distances were plotted to visualize the sequence space occupied by TBEV (Figure 2 and Figure 3). Each sequence pair in a dataset was represented by a dot with coordinates reflecting the nucleotide and amino-acid sequence distance between these sequences. The density of possible distance values was indicated by color (Figure 2). Using a heatmap plot in pairwise nucleotide/amino-acid distance coordinates from one hand showed the density; from the other, such a demonstration was possible by summarizing all dots in a picture element. This led to the “averaging” of real data. To reveal the level of such averaging, all virus pairs were divided into intrasubtype pairs (viruses from the same subtype, indicated by the red color at Figure 3) and intersubtype pairs (viruses from different subtypes, indicated by the blue color at Figure 3). Furthermore, these pairs were plotted with appropriate coordinates without any averaging. Intersubtype pairs were indicated by blue circles, while intrasubtypes were indicated by red circles (Figure 3). The overall situation has not changed in comparison to heatmap plot (Figure 2, two upper panels).

Uncorrected distances were used to provide consistent conclusions and reduce the effect of sample bias. All intersubtype virus pairs between three major subtypes (Sib, Eur, and FE) had nucleotide (nt) distances above 10% (Figure 2, *x*-axis, third panel), whereas all intrasubtype distances were below 10% (Figure 2, three bottom panels). There was a very clear border between inter- and intrasubtype nucleotide sequence distances, as none of the 20,910 ORF sequence pairs had distances in the range of 10–13% and none of the 183,921 *E* gene sequence pairs had values between 10% and 11% (Figure 2, third panel). Just one pair of viruses did not meet the proposed criterion when fragments of the *E* gene were used. #KT321397 and #MK284389 both belonged to TBEV-Sib but differed in 10.02% of nt (105 out of 1030 nt) in the analyzed *E* gene fragment (black arrow at bottom panel at Figure 3). Amino-acid distances of the E protein (Figure 2, *y*-axis, left part of the third panel) did not distinguish major subtypes. Full ORF amino-acid sequence distances distinguished major TBEV subtypes with few overlaps (Figure 2, *y*-axis, right part of the third panel).

Across all TBEV sequences (three major types and novel provisional subtypes), intersubtype nucleotide distances were above the 10% threshold (dotted line at Figure 2 and Figure 3), whereas intrasubtype virus pairs were below. This pattern was observed both for full ORF sequences and for the *E* gene fragment. In the latter case, the separation was not ideal, but the overlap between inter- and intrasubtype distances was produced solely by two “2871” group representatives. Omitting them resulted in clear segregation of inter- and intrasubtype distances, even in the E protein fragment (two upper panels at Figure 2 and Figure 3).

The 10% nucleotide sequence distance threshold clearly supported the segregation of four provisional novel subtypes (Figure 1) as distinct entities. Similar to the three major subtypes, amino-acid sequence distances could not unambiguously distinguish seven provisional TBEV subtypes in the *E* gene (two upper panels at Figure 2 and Figure 3, *y*-axis). In the full ORF, the number of overlaps involving amino-acid sequences of putative new subtypes (Figure 2, two upper panels) was higher than among the three major types (Figure 2, third panel); thus, amino-acid sequences cannot be recommended as a subspecies criterion in TBEV.

## 4. Discussion

Currently, species are the lowest taxonomic level in the viral hierarchy approved by ICTV. Yet, lower taxonomic levels do not have a standard designation. TBEV groups below the species level may be called “subtype” [7], “lineage” [38,39], or “genotype” [40,41]. These terms indicate the same entity. Of these, “subtype” is the most widely used term. As of August 2020, Scopus searches with keywords “TBEV AND subtype” yielded 161 papers, “TBEV AND genotype” yielded 47, and “TBEV AND lineage” yielded 25. Therefore, the statistics of traditional usage suggests using the term “subtype” for the designation of the first taxon below TBEV species. TBEV subtype abbreviations are also not unified (Table 2). Considering the most common usage and reasonable unification, TBEV-Eur can be suggested to abbreviate the European subtype, with TBEV-Sib for the Siberian subtype, TBEV-FE for the Far-Eastern subtype, TBEV-Bkl-1 for 178–79 (“genotype 4”), TBEV-Bkl-2 for 886–84 (“genotype 5”), and TBEV-Ob for 2871 (“Obskaya lineage”) (Table 2).

The distribution of all possible TBEV pairwise distances indicated that the three major subtypes were clearly distinguished by a 10% nucleotide sequence threshold. Applying this threshold to prospective subtypes supported their segregation as distinct subtypes, not variants of the three major types. Thus, according to this simple nucleotide distance cutoff, known representatives of the TBEV species may be divided into seven subtypes.

TBEV subtypes could be perfectly distinguished by nucleotide sequences but not so well by amino-acid sequences. Several important conclusions follow from this observation. A higher rate of synonymous substitutions [68] suggests that the selection pressure in TBEV is mainly stabilizing [69]. Nucleotide sequence distances, both within and between subtypes, were below the level of synonymous mutation saturation (about 20%). This is concordant with the hypothesis that *TBEV* is a rather “young” virus that emerged recently, 1000–10,000 years ago, according to various estimates of substitution rates [13,24,50,70].

It is not unlikely that our knowledge of TBEV sequences remains limited (e.g., rare variants remain undiscovered), but the remarkably stark separation of subtypes observed so far requires an explanation. If the virus was gradually changing over time, there would have been a smooth gradient of genetic distances. The actual bimodal distribution of nucleotide distances can be explained by either (1) quantum events (rapid adaptation of a subtype to a new host or niche, possibly a factor for TBEV-Eur that has its distinct vector) or (2) the relatively long persistence of a virus in a limited focus and subsequent extinction of intermediate lineages and global spread of the few contemporary ones, or a combination of the two mechanisms.

The protein E encoding region is historically the most commonly sequenced TBEV genome fragment. However, the analysis of pairwise distances among TBEV representatives suggested that it has evolutionary patterns distinct from most other genomic regions. In the complete ORF, there was a correlation between nucleotide and amino-acid distances, suggesting that their accumulation was ruled by similar mechanisms (random mutation and fixation). In the *E* gene, there were examples of remarkable conservation in amino-acid sequences, even between distinct subtypes. For example, #KT001070 (TBEV-FE) and #MH481365 (TBEV-Bkl-2) differed by 13% of nucleotides (134 out of 1030 nt) and 0.3% of amino acids (one out of 343 aa). Thus, even minor amino-acid substitutions involving similar amino acids were purged by negative selection. This observation implies that stabilizing selection and random sorting, rather than adaptation, could be the evolutionary force shaping TBEV subtypes. Notably, such negative selection was much less pronounced in the full ORF compared to *E* gene. For example, #KT001070 and #MH481365 differed by 12% of nucleotides (1289 out of 10,245 nt) and 4% of amino acids (124 out of 3414 aa).

Some microevolutionary studies suggested that the TBEV E protein coding gene may change more rapidly than other genome regions [71]. At the same time, on the amino-acid level, it turned out to be one of the most conserved TBEV proteins. The E protein is responsible for binding to host receptors [72] and largely influences TBEV serology. A high level of E protein sequence conservation between subtypes means that TBEV subtypes are not necessarily associated with serological properties and herd immunity is an unlikely evolutionary pressure force in subspecies differentiation of the virus. Indeed, pronounced cross-reactivity between TBEV subtypes supports this notion [73].

The discrete distribution of traits within a species, including genetic distance, is not natural. The 10% subtype cutoff is a convenient number today, but it is unlikely to persist for a prolonged time due to both accumulation of substitutions in circulating viruses and the discovery of novel viruses. The first possibility is exemplified by two viruses belonging to the TBEV-Sib subtype but differing in 10.02% of the *E* gene fragment (arrow in Figure 3).The latter possibility is highlighted by the discovery of divergent TBEV-Eur variants in The Netherlands [47] and United Kingdom [65], which extended the known nucleotide sequence variation within the TBEV-Eur subtype from around 3% to 9% in the ORF region.

## 5. Conclusions

Herein, all viruses belonging to the species *TBEV* were investigated in nucleotide/protein divergence coordinates. Whenever the ORF of two viruses differed by less than 10% nucleotide sequence, these viruses belonged to the same subtype. According to this cutoff, suggested as the subtype border, *TBEV* species can be divided into seven subtypes: TBEV-Eur, TBEV-Sib, TBEV-FE, TBEV-Ob (TBEV-2871), TBEV-Him, TBEV-Bkl-1 (178–79), and TBEV-Bkl-2 (886–84).

## Figures and Tables

**Table 1:** Number of known nonidentical sequences for provisional TBEV subtypes.

Subtype Name	*E* gene Fragment Sequences	ORF Sequences
TBEV-Eu	242	73
TBEV-Sib	295	40
TBEV-FE	133	92
TBEV-Him	2	2
TBEV-Bkl-1 (178-79)	1	1
TBEV-Bkl-2 (886-84)	9	7
TBEV-Ob	2	1

**Table 2:** Variants of TBEV subtype abbreviations.

**European Subtype**	
TBEV-Eu	[24,42,43,44,45,46]
TBEV-EU	[47,48,49]
TBEV-Eur	[50,51,52,53,54,55]
W-TBEV	[56,57]
Eu-TBEV	[23,25,58]
**Siberian Subtype**	
Sib-TBEV	[23,25]
TBEV-Sib	[24,36,44,45,46,48,50,52]
TBEV-S	[59,60]
S-TBEV	[11,57,58]
**Far-Eastern Subtype**	
TBEV-FE	[16,24,42,45,48,50,52]
TBEV-Fe	[44,46,61,62,63]
FE-TBEV	[23,25,56,57,64]
**“178-79”, or “Genotype 4”**	
Genotype 4	[18]
strain 178-79	[23]
“178-79” strain	[22]
**“886-84”, or “Genotype 5”, or “Baikalian Subtype”**	
Genotype 5	[18]
TBEV-Bkl	[21]
TBEV-Blk	[65]
TBEV-B	[20]
“group 886”	[23]
“886-84-like” strains	[22]
**Himalayan Subtype**	
Him-TBEV	[25,66]
TBEV-Him	[45,67]
**2871 (“Obskaya Lineage”)**	
Obskaya lineage; TBEV-2871 strain	[13,24]
